# An intronic RNA structure modulates expression of the mRNA biogenesis factor Sus1

**DOI:** 10.1261/rna.054049.115

**Published:** 2016-01

**Authors:** Ali AbuQattam, José Gallego, Susana Rodríguez-Navarro

**Affiliations:** 1 Gene Expression and RNA Metabolism Laboratory, Centro de Investigación Príncipe Felipe, Valencia 46012, Spain; 2 Facultad de Medicina, Universidad Católica de Valencia, Valencia 46001, Spain

**Keywords:** Sus1, gene expression, splicing, yeast, NMR, RNA, structure, thermal stability

## Abstract

Sus1 is a conserved protein involved in chromatin remodeling and mRNA biogenesis. Unlike most yeast genes, the *SUS1* pre-mRNA of *Saccharomyces cerevisiae* contains two introns and is alternatively spliced, retaining one or both introns in response to changes in environmental conditions. *SUS1* splicing may allow the cell to control Sus1 expression, but the mechanisms that regulate this process remain unknown. Using in silico analyses together with NMR spectroscopy, gel electrophoresis, and UV thermal denaturation experiments, we show that the downstream intron (I2) of *SUS1* forms a weakly stable, 37-nucleotide stem–loop structure containing the branch site near its apical loop and the 3′ splice site after the stem terminus. A cellular assay revealed that two of four mutants containing altered I2 structures had significantly impaired *SUS1* expression. Semiquantitative RT-PCR experiments indicated that all mutants accumulated unspliced *SUS1* pre-mRNA and/or induced distorted levels of fully spliced mRNA relative to wild type. Concomitantly, Sus1 cellular functions in histone H2B deubiquitination and mRNA export were affected in I2 hairpin mutants that inhibited splicing. This work demonstrates that I2 structure is relevant for *SUS1* expression, and that this effect is likely exerted through modulation of splicing.

## INTRODUCTION

Introns are removed from pre-mRNAs to create a mature transcript by the action of the splicing machinery (Wahl et al. 2009; Fica et al. 2013). Recent reports have shown that splicing contributes to the control of gene expression (Acuña and Kornblihtt 2014; Moehle et al. 2014) and can “fine-tune” different stages of the gene expression process by regulating the expression levels of key pathway components involved in RNP and ribosome biogenesis, transcription, and RNA transport (Johnson and Vilardell 2012). Some of these studies have focused on *Saccharomyces cerevisiae* Sus1, a factor involved in transcription and mRNA export (Rodríguez-Navarro et al. 2004; Galan and Rodríguez-Navarro 2012). Sus1 is part of the SAGA (Spt–Ada–Gcn5-Acetyltransferase) complex where it participates as a modulator of the deubiquitinase activity of Ubp8 (Köhler et al. 2006). It also interacts at the nuclear pore with TREX-2 (transcription export complex 2) and participates in mRNA export and genome stability (González-Aguilera et al. 2008). In addition, Sus1 transiently interacts during transcription elongation with RNA polymerase II and the export factors Yra1 and Mex67 (Pascual-García et al. 2008). An interesting feature of the *SUS1* gene—highly infrequent in the *S. cerevisiae* genome—is the fact that it contains two introns (Rodríguez-Navarro et al. 2004). The presence of two introns in the *SUS1* gene opens up the possibility of a feedback mechanism involving splicing. A similar situation was found in the *YRA1* gene encoding another essential mRNA export factor, whose expression was found to be controlled by different mRNA biogenesis steps (Rodríguez-Navarro et al. 2002; Preker and Guthrie 2006; Dong et al. 2007).

The functional relevance of the presence of two introns in the *SUS1* pre-mRNA has been studied previously (Cuenca-Bono et al. 2011; Hossain et al. 2011). While intron 2 (I2) has consensus splice sequences, intron 1 (I1) is characterized by the presence of nonconsensus 5′ splice site (SS) (GUAUGA instead of canonical GUAUGU) and branch site (BS) (UACUGAC instead of UACUAAC). These sequences influence *SUS1* splicing and as a consequence intron 1 is retained in >15% of the *SUS1* transcripts. Moreover, retention of I1 is affected by growth conditions, suggesting a potential role for splicing in regulating Sus1 cellular function (Johnson and Vilardell 2012).

RNA structure is emerging as an important element in the regulation of gene expression, via modulation of interactions with proteins and microRNAs or by arranging the spatial distribution of important sequences (Wan et al. 2015). For example, in the case of splicing intron RNA folding has been shown to influence 3′SS selection through the masking of AG sequences inside double-helical stems, or by bringing BS and 3′SS sequences into functional proximity (Deshler and Rossi 1991; Rogic et al. 2008; Warf and Berglund 2010; Meyer et al. 2011; Pérez-Valle and Vilardell 2012). In *S. cerevisiae*, intronic RNA structures have been reported to play a role in autoregulating *RPL30* and *YRA1* expression at different levels (Dong et al. 2010; Meyer et al. 2011). Gaining insight into the role of RNA structure in controlling mRNA biogenesis at different stages is a major challenge in the field. In this article, we explore the regulatory role of a stem–loop structure formed by I2 of *SUS1*.

## RESULTS

### Intron 2 of *SUS1* forms a weakly stable hairpin structure in solution containing BS nucleotides in its apical loop

**FIGURE 1.**
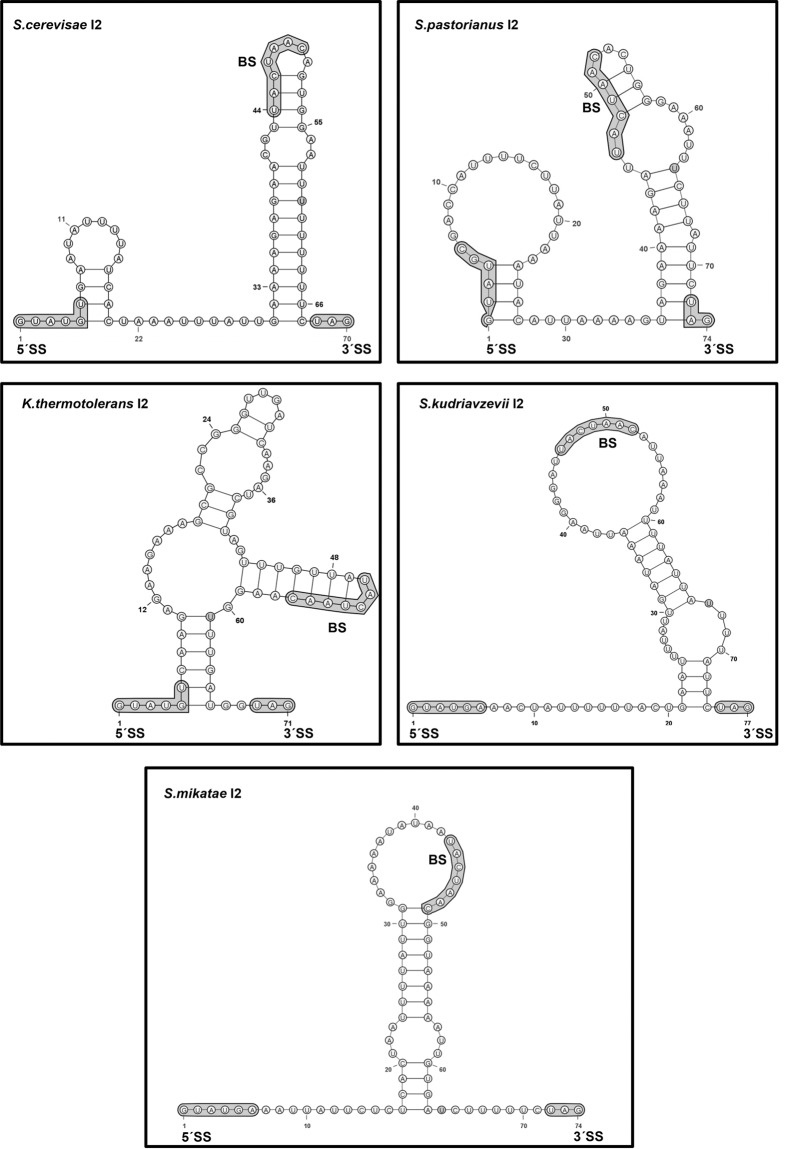
Predicted secondary structure of *SUS1* intron 2 RNA. Minimum free-energy I2 structures in *S. cerevisiae, S. pastorianus, K. thermotolerans, S. kudriavzevii*, and *S. mikatae*. The 5′SS, 3′SS, BS sequences and the uridine at −9 position preceding the AG are highlighted. The predictions were carried out with Mfold.

The RNA-folding algorithm Mfold (Zuker 2003) predicted that I2 of *S. cerevisiae SUS1* formed a 37-nucleotide (nt) stem–loop structure with BS nucleotides lying in the apical loop and 3′SS nucleotides immediately after the 3′ stem terminus (Fig. 1; Hossain et al. 2011). The pre-mRNAs of several yeast species, selected from our previous evolutionary analysis of *SUS1* (Cuenca-Bono et al. 2011), were also subjected to secondary structure predictions. The analyses revealed comparable putative hairpins formed by the second intron of *S. pastorianus, K. thermotolerans, S. kudriavzevii,* and *S. mikatae*. In these hairpins, the BS nucleotides similarly localized close to the apical loop, and the 3′SS near the 3′ terminus of the base-paired stem (Fig. 1). These results were generally consistent with previous predictions in silico (Hossain et al. 2011).

**FIGURE 2.**
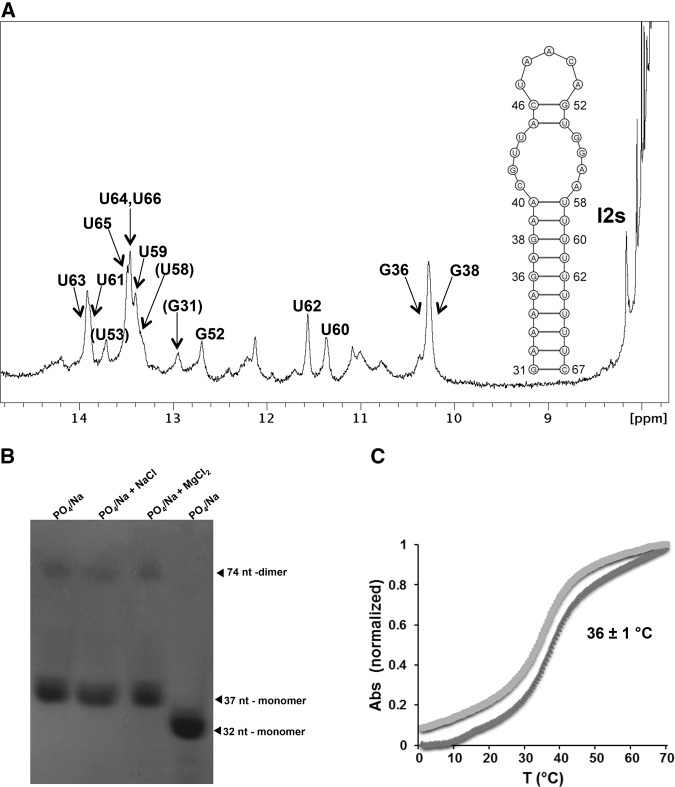
In vitro analysis of intron 2 structure and stability. (*A*) Imino proton NMR spectrum and NMR-supported secondary structure of the 37-nt I2s oligomer. Conditions: 7°C, 2 mM sodium phosphate (pH 6.0), and 100 mM NaCl. The assignments (parentheses indicate tentative assignments) were obtained from NOESY and TOCSY spectra in H_2_O and D_2_O. (*B*) Native gel comparing the electrophoretic mobility of 16 µM I2s samples previously annealed in different ionic conditions: (i) 2 mM sodium phosphate (pH 6.0), (ii) 2 mM sodium phosphate and 100 mM NaCl, (iii) 2 mM sodium phosphate, and 2 mM MgCl_2_. Lane *4* contained a 32-nt RNA hairpin control. (*C*) UV-monitored thermal denaturation curve of I2s in 2 mM sodium phosphate (pH 6.0) and 100 mM NaCl. The average melting temperature of I2s under these ionic conditions is indicated in the graph.

To confirm the computational predictions, we next examined by NMR spectroscopy and gel electrophoresis the conformation of *S. cerevisiae* I2, represented by a 37-nt oligomer (hereafter named I2s) encompassing the hairpin predicted to be formed by I2 nucleotides 31–67 (Fig. 2). The electrophoretic results indicated the formation of a predominantly monomeric structure in different solution conditions. The NMR data revealed that I2s adopted the hairpin structure depicted in Figure 2. The presence of the A_32_AAAGAGAA_40_:U_58_UUUUUUUU_66_ double-helical tract at the base of the hairpin was demonstrated by NOE interactions involving exchangeable and nonexchangeable resonances. The two G36:U62 and G38:U60 wobble pairs were clearly established by the characteristic chemical shift and NOE pattern of the G H1 and U H3 iminos (Fig. 2A; Supplemental Fig. S1A, crosspeaks a and b). Likewise, diagnostic A H2–U H3 interactions allowed identification of eight Watson–Crick A:U pairs in the I2s hairpin (Fig. 2A; Supplemental Fig. S1B). A35:U63, A37:U61, and A39:U59 were assigned from NOE contacts involving protons of G36:U62 and G38:U60 (Supplemental Fig. S1A, crosspeaks c–g, i, and j; Supplemental Fig. S1C, crosspeaks k, l, n, and q). The remaining four A:U pairs of the lower stem were identified by cross-strand A H2-U H3 NOEs (indicated by solid horizontal arrows in Supplemental Fig. S1B), as well as by sequential intra-strand and cross-strand H2-H1′ NOEs typical of canonical RNA stems (Supplemental Fig. S1C).

The NMR data were also consistent with the presence of the two Watson–Crick pairs forming the A_45_C_46_:G_52_U_53_ upper stem below the apical loop. The A45:U53 pair was suggested by the remaining A H2–U H3 crosspeak, whose resonances were not connected by NOE interactions with the remaining A:U pairs of the system (Supplemental Fig. S1B,C). Diagnostic crosspeaks between guanine imino and cytosine amino protons that were unrelated to the terminal C67 residue allowed identification of the C46:G52 pair (Supplemental Fig. S1A, crosspeak h). There were additional imino resonances in the I2s spectrum (Fig. 2A) whose chemical shifts were consistent with the formation of G:U pairs in the CGUU:GGAA internal loop separating the two canonical stems, but they exchanged too quickly with the solvent to be assigned.

The stability of the hairpin formed by intron 2 of *SUS1* was evaluated with UV-thermal denaturation experiments. The results indicated that this stem–loop structure had low thermal stability: the I2s hairpin melted at 26°C in the absence of NaCl and at 36°C with 100 mM NaCl, in ionic conditions approximately similar to those found in a cellular environment. The thermal denaturation curves were reversible in all cases (Fig. 2C; Supplemental Fig. S2).

### Partial deletion of the stem–loop structure formed by intron 2 modulates *S. cerevisiae SUS1* splicing

**FIGURE 3.**
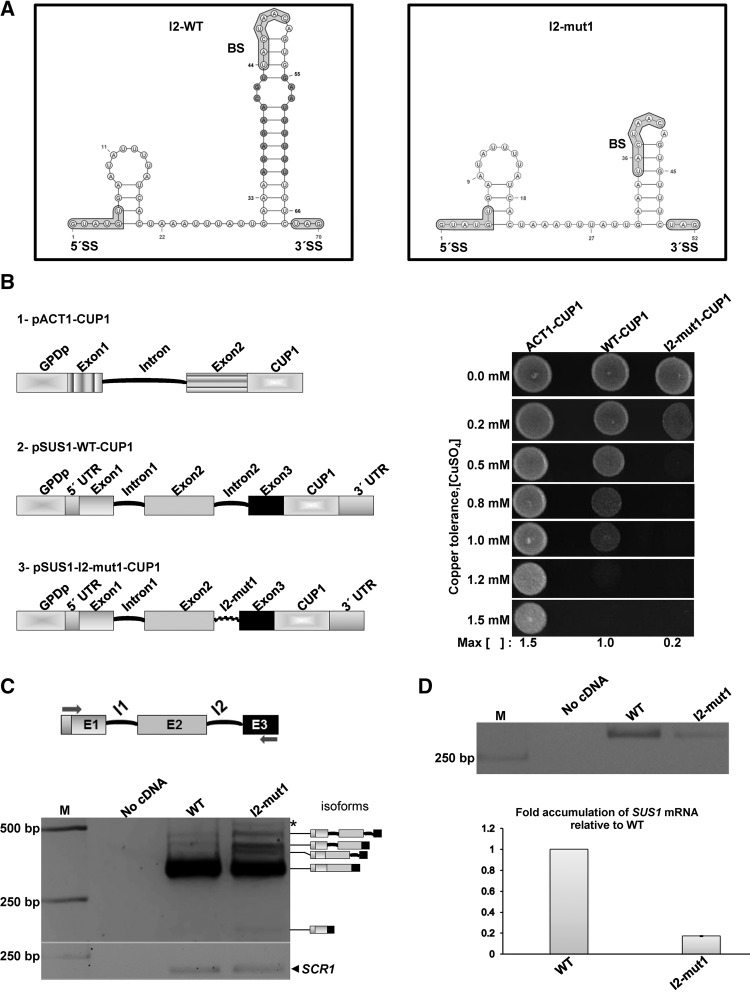
Partial deletion of the I2 hairpin double-helical stem affects *SUS1* splicing. (*A*) MFold-predicted secondary structure of wild-type I2. The nucleotides highlighted in dark gray were removed to construct the intron 2 deletion mutant 1 (I2-mut1). The prediction of I2-mut1 secondary structure is shown on the *right*. (*B*) Copper assay of *cup1*Δ cells transformed with plasmids containing SUS1g-CUP1, SUS1-I2-mut1-CUP1, and ACT1-CUP1 as a control. Maximum copper tolerance is indicated. (*C*) Semi-q-RT-PCR (35 cycles) to amplify *SUS1* transcripts resolved in a 3% agarose gel from cells expressing SUS1g-CUP1 (WT) or SUS1-I2-mut1-CUP1 (I2-mut1) in each case. A PCR without cDNA (no cDNA) was included as control. The stick diagrams on the *right* of the gel show the different isoforms of *SUS1* RNA. (*D*) Semi-q-RT-PCR (25 cycles) to amplify *SUS1* transcripts resolved in a 3% agarose gel from cells expressing SUS1g-CUP1 (WT) or SUS1-I2-mut1-CUP1 (I2-mut1) in each case (*upper* panel). qPCR showing mRNA accumulation from cells expressing SUS1g-CUP1 (WT) or SUS1-I2-mut1-CUP1 (I2-mut1) normalized to the amount of *SCR1*ncRNA (*lower* panel). Error bars represent SE for at least three independent experiments ([*] Spurious PCR product).

The presence of a hairpin structure formed by I2 of *SUS1* in *S. cerevisiae* together with its conservation in several yeast species suggested a possible functional relevance. To address this question, we studied an I2 deletion mutant (I2-mut1) in which the double-helical stem of the predicted hairpin was shortened by 9 base pairs (bp) while the BS was maintained in the apical loop and the 3′SS after the stem (Fig. 3A). This mutant had 18 nt less relative to wild-type I2, but all intronic features relevant for splicing were well within functional range: the total size of the mutant intron was 52 nt (the minimum size has been proposed to be 50 nt), the distance between the 5′SS and the BS was 28 nt (greater than the required 25 nt minimum), and there were 12 nt between the BS and the 3′SS (10 nt are necessary in this case as a minimum) (Meyer et al. 2011; Pérez-Valle and Vilardell 2012). To evaluate the expression efficiency of this mutant in vivo, we used the *CUP1* reporter system (Lesser and Guthrie 1993). *cup1*Δ cells were transformed with one of the following reporter constructs: pACT1-CUP1, pSUS1g-CUP1 (containing a *SUS1* gene with wild-type I1 and I2 introns), and pSUS1-I2-mut1-CUP1 (with a wild-type I1 and a shortened I2 hairpin). Splicing was monitored both by assessing copper tolerance and by semiquantitative reverse transcription PCR (semi-qRT-PCR). Partial deletion of the stem of the I2 hairpin led to a clear reduction in copper tolerance compared with wild-type I2 (Fig. 3B), suggesting that the I2 hairpin was important for *SUS1* expression. When the different *SUS1* splice forms generated from expressing I2-mut1 in *sus1*Δ cells were measured by semi-qRT-PCR, the accumulation of different pre-mRNAs containing I2, I1 or both introns was evident (Fig. 3C). In agreement with the results of the copper assay, semi-qRT-PCR experiments indicated a reduced amount of fully spliced mRNA relative to the wild-type cultures (Fig. 3D). Since I1-containing isoforms also accumulated with the I2-mut1 mutant (Fig. 3C), we generated an I2-mut1 construct lacking I1 (I2-mut1ΔI1). The absence of I1 did not change the effect of I2-mut1 on *SUS*1 processing as evidenced by cell growth in copper-containing plates (Supplemental Fig. S3). This demonstrated that the splicing effects caused by the I2 mutation did not depend on I1.

A fast-migrating *SUS1* transcript was detected in the semi-qRT-PCR gels of I2-mut1 (Fig. 3C, last lane). Sequencing of this band revealed that it corresponded to the *SUS1* E1–E3 transcript, which was already observed (Hossain et al. 2011). Partial deletion of the double-helical stem of the I2 hairpin promoted accumulation of I1- and I2-containing pre-mRNAs (Fig. 3C). The poor splicing efficiency of I2-mut1 could have led to increased exon 2 skipping, which contributes to alternative splicing and the production of the E1–E3 isoform (discussed below).

### Stabilization of the intron 2 hairpin affects *SUS1* expression

**FIGURE 4.**
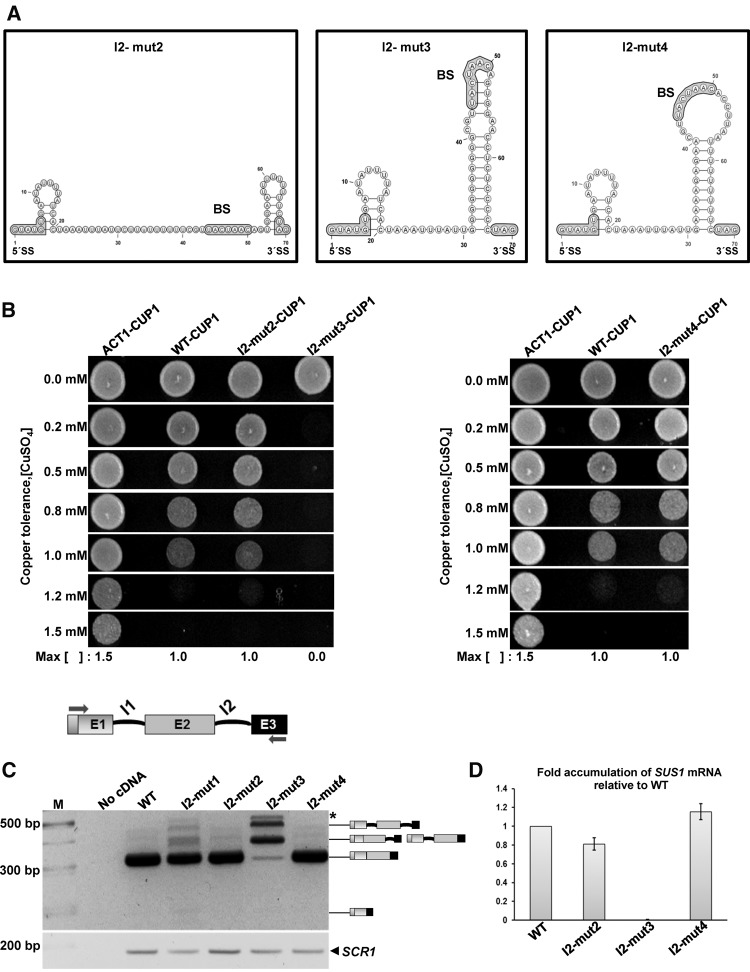
Stabilization of the intron 2 hairpin affects *SUS1* expression. (*A*) MFold-predicted secondary structure of the I2 hairpin disruption mutant (I2-mut2, *left* panel), hairpin stabilization mutant (I2-mut3, *middle* panel) and BS base-pairing disruption mutant (I2-mut4, *right* panel). The nucleotides highlighted in gray were substituted to generate each mutant. (*B*) Copper assay of *cup1*Δ cells transformed with plasmids containing SUS1g-CUP1, SUS1-I2-mut2-CUP1, SUS1-I2-mut3-CUP1, SUS1-I2-mut4-CUP1, and ACT1-CUP1 as a control. Maximum copper tolerance is indicated. (*C*) Semi-q-RT-PCR (35 cycles) to amplify *SUS1* transcripts resolved in a 3% agarose gel from cells expressing SUS1g-CUP1 (WT) or SUS1-I2-mut1-CUP1 (I2-mut1), SUS1-I2-mut2-CUP1 (I2-mut2), SUS1-I2-mut3-CUP1 (I2-mut3), and SUS1-I2-mut4-CUP1 (I2-mut4) in each case. A PCR without cDNA (no cDNA) was included as a control. The stick diagrams on the *right* of the gel show the different isoforms of *SUS1* RNA. (*D*) qPCR showing mRNA accumulation from cells expressing *SUS1* as in (*C*) normalized to the amount of *SCR1* ncRNA. Error bars represent SE for at least three independent experiments ([*] Spurious PCR product).

The functional relevance of the I2 hairpin was further studied by analyzing three new structural mutants. Mutant I2-mut2 was designed to disrupt the 10-bp stem at the base of the I2 hairpin by replacing the 10-nt G_31_AAAAGAGAA_40_ purine stretch with C_31_UUUUUUUUU_40_ (Fig. 4A, left panel). In contrast, I2-mut3 was intended to stabilize this 10-bp stem by converting its seven A:U pairs into G:C pairs: thus the As of the G_31_AAAAGAGAA_40_ purine stretch were replaced with Gs, and the opposing Us in the U_58_UUUUUUUUC_67_ pyrimidine segment (U_58_–U_59_, U_61_, and U_63_–U_66_) with Cs (Fig. 4A, middle panel). On the other hand, I2-mut4 was aimed to disrupt the base pairs affecting BS nucleotides without altering the lower double-helical stem of the hairpin. To achieve this, the G_52_UGG_55_ segment was replaced with C_52_CUU_55_ (Fig. 4A, right panel). Note that none of these I2-mut2, I2-mut3, or I2-mut4 mutants affected the size of the intron, the number of nucleotides separating the BS and the 3′SS, or the PPT following the BS. The effect of these mutants on the structure and stability of the I2 hairpin was assessed with Mfold calculations (Fig. 4A; Supplemental Fig. S4A) as well as with gel electrophoresis and thermal denaturation analyses of mutant 37-nt I2s oligonucleotides (Supplemental Fig. S4B,C). In the predicted structure of I2-mut3, G:C pairs replaced the seven A:U pairs of the stem at the base of the I2 hairpin. In agreement with this, the I2-mut3s oligomer formed a highly stable monomeric structure with a *T*_m_ value of 86°C (Supplemental Fig. S4B), more than two times higher than the *T*_m_ of wild-type I2s, which melted at 36°C in the same ionic conditions (Fig. 2). In the predicted structure of the full-length I2-mut2 intron the 10-bp lower stem was completely eliminated and the BS lied in a fully unstructured region (Fig. 4A). Although the PPT nucleotide formed a small hairpin whose terminal pairs partially encompassed the 3′SS, these terminal U:G and A:U pairs showed significant positional entropy in the ensemble of theoretical structures (data not shown) and are likely unstable. In agreement with these predictions, the I2-mut2s oligonucleotide gave rise to delayed electrophoretic bands and did not exhibit any detectable transition in UV-monitored thermal denaturation experiments (Supplemental Fig. S4), indicating the formation of a very unstable structure or no structure at all. In the I2-mut4 predicted fold, the BS nucleotides did not form any base pairs in the apical region of the hairpin while the AU-rich lower stem remained unaffected (Fig. 4A; Supplemental Fig. S4A). In accordance with this, I2-mut4s formed a monomeric structure with a slightly reduced *T*_m_ value relative to wild-type I2s (32°C versus 36°C in the presence of 100 mM NaCl) (Supplemental Fig. S4).

We then evaluated the impact of each of these structural mutants on *SUS1* expression as described above for I2-mut1. The I2-mut2, I2-mut3, and I2-mut4 mutants were first cloned in the *CUP1* reporter system and transformed into *cup1*Δ cells. As shown in Figure 4B, left panel, I2-mut3 drastically impaired cell growth relative to wild type, indicating that stabilization of the I2 stem–loop decreased *SUS1* expression. In contrast, I2-mut2 and I2-mut4 did not impair cell growth when compared to wild type (Fig. 4B, left and right panels, respectively). To assess the production of *SUS1* isoforms by these mutants, semi-q-RT-PCR was carried out to amplify *SUS1* transcripts in *sus1*Δ cells bearing mutant constructs. In agreement with the copper assay observations, the I2-mut3 mutant stabilizing the stem–loop structure completely inhibited I2 splicing, leading to a clear enrichment of I2- and I1-I2-containing pre-mRNA *SUS1* transcripts (Fig. 4C). The I2-mut2 and I2-mut4 mutant sequences did not lead to a detectable increment of I1 or I2 retention in the gels (Fig. 4C), but semi-q-RT-PCR analyses of I2-mut2 transcripts showed a slight reduction of fully spliced *SUS1* mRNA relative to wild type (Fig. 4D), while a small increase was observed in the case of I2-mut4 (Fig. 4D).

### High-temperature effects on *SUS1* splicing upon intron 2 structural mutations

**FIGURE 5.**
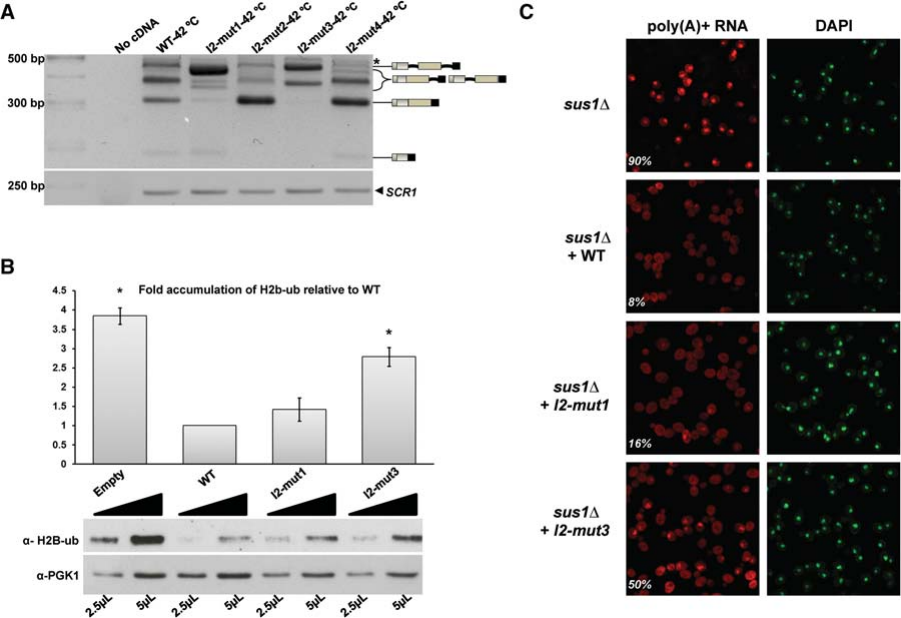
Deletion or stabilization of intron 2 hairpin affects Sus1 cellular roles. (*A*) Analysis of *SUS1* transcripts by semi-q-RT–PCR from cells expressing SUS1g (WT) or I2 structural mutants (SUS1-I2-mut1-CUP1 (I2-mut1), SUS1-I2-mut2-CUP1 (I2-mut2), SUS1-I2-mut3-CUP1 (I2-mut3), and SUS1-I2-mut4-CUP1 (I2-mut4) from cells incubated at 42°C for 20 min. Bands corresponding to the different transcripts are indicated. A PCR without cDNA (no cDNA) was included as control. (*B*) Quantification and representative blots obtained with increasing concentration of whole-cell extracts (WCE) from *sus1*Δ cells transformed with an empty plasmid (Empty), or with plasmids expressing SUS1g (WT), SUS1-I2-mut1 (I2-mut1), or SUS1-I2-mut3 (I2-mut3). Two volumes (2.5 µL or 5 µL) of each WCE were resolved by 15% SDS-polyacrylamide gel, transferred to a nitrocellulose membrane. The membrane was cut and the upper part was incubated with *PGK1* antibody (loading control) and the lower with H2B-ub antibody. Bar graph shows total H2B-ub levels normalized to *PGK1* after quantification of Western blot signals. Similar results were obtained using total H2B as a loading control (data not shown). Error bars represent SE for at least three independent experiments. (*C*) Representative images of poly(A)^+^ RNA localization in *sus1*Δ cells, transformed as in (*B*) and assayed by in situ hybridization using Cy3-labeled oligo(dT) probes. The percentages indicate the number of cells showing mRNA accumulation and correspond to the averages of at least three independent biological replicates. DAPI staining to mark the nucleus is shown on the *right*. Cells were incubated at 37°C in rich media for 2 h.

Interesting differences were observed between the structural mutants when the experiments were carried out under stress. Previous analyses with wild-type cells incubated at 42°C revealed reduction of *SUS1* expression, accompanied by accumulation of unspliced *SUS1* transcripts and decrease of fully spliced mRNA (Cuenca-Bono et al. 2011). We tested the effect of the different structural mutants in these conditions by semi-q-RT-PCR (Fig. 5A). The most drastic variation relative to standard conditions (cells growing at 30°C) was observed for mutants I2-mut1 and I2-mut3, which, compared with the wild-type sequence, generated a large accumulation of transcripts containing both introns, together with a drastic reduction of fully spliced mRNA. At 42°C, I2-mut2 and I2-mut4 exhibited less intron retention as well as increased levels of fully spliced mRNA relative to wild type. In addition, I2-mut2 clearly showed less I1 retention relative to wild type or I2-mut4 (Fig. 5A). This confirmed that I2 splicing can affect an upstream splicing step, supporting the results obtained with mutant I2-mut1.

### Sus1 functions in histone deubiquitination and mRNA export are affected in I2 hairpin mutants that inhibit splicing

Sus1 is a conserved factor that is required for mRNA export and histone H2B deubiquitination, and deletion or mutation of *SUS1* has been shown to impair mRNA export and histone H2B deubiquitination (Rodríguez-Navarro et al. 2004; Köhler et al. 2006; Kurshakova et al. 2007; Zhao et al. 2008; Klöckner et al. 2009; Kopytova et al. 2010; Galan and Rodríguez-Navarro 2012; García-Oliver et al. 2012). So far, our analyses have shown that partial deletion (I2-mut1) and stabilization (I2-mut3) of the 37-nt hairpin structure formed by intron 2 led to differential *SUS1* isoform expression. These changes in splicing could lead to a deregulation of Sus1 protein production. To study whether these I2 mutations had functional consequences, cells lacking the *SUS1* gene (*sus1*Δ) were transformed with wild-type and I2-mutant *SUS1* plasmids, and their ability to complement *SUS1* deletion phenotypes was evaluated. As shown in Figure 5B, I2-mut3 was unable to deubiquitinate histone H2B at wild-type levels, in agreement with the low expression of fully spliced *SUS1* isoform in these cells (Fig. 4C). Although I2-mut1 likewise reproducibly reduced the amount of fully spliced *SUS1* (Fig. 3C,D), the reduction of ubiquitinated H2B relative to WT was not statistically significant (Fig. 5B). Similar results were obtained using total H2B as a loading control (data not shown). Concomitantly, defects in mRNA export provoked by *SUS1* deletion observed in 90% of the cells (Fig. 5C, left panel) were more apparent for I2-mut3 than I2-mut1: 50 and 16% of the cells transformed with these two mutants, respectively, accumulated mRNA, relative to 8% transformed with wild-type *SUS1* (Fig. 5C).

## DISCUSSION

The structure of RNA molecules plays an important role during their metabolism, adding a new layer of regulation to different steps of the gene expression pathway. In this study, we have analyzed the impact on splicing of a hairpin structure formed by the second intron of the pre-mRNA of *S. cerevisiae SUS1*. Secondary structure predictions of this intron in this and other yeast species revealed the formation of a stem–loop structure with BS nucleotides located near the apical loop and 3′SS nucleotides close to the 3′ terminus of the base-paired stem (Fig. 1). In several cases, 5′SS nucleotides were likewise predicted to form base pairs with downstream nucleotides that might affect splicing efficiency. However, our study focused on the hairpin formed by the BS and 3′SS segment, leaving the 5′SS region unmodified. In all species in which the I2 hairpin was present a pyrimidine-rich sequence was located between the BS and the 3′SS. This polypyrimidine tract (PPT) has been shown to be important for splicing in higher eukaryotes (Roscigno et al. 1993; Liu and Mertz 1996; Coolidge et al. 1997). In contrast, in yeast species the 5′SS, BS, and 3′SS sequences are highly conserved, but the PPTs are not (Rymond and Rosbash 1985; Fouser and Friesen 1987). Interestingly, the PPT of *SUS1* I2 seemed to be partially conserved in yeast, and a uridine at position –9 preceding the 3′SS was present in all cases (Fig. 1). It has been suggested that splicing in fungi may be different from that in vertebrates and may require additional proteins that interact with PPTs upstream of the BS (Kupfer et al. 2004). However, other studies have indicated that PPTs downstream from the BS do provide a positioning function (Matlin et al. 2007). Furthermore, nonconserved sequences can also influence alternative splice site selection via structural motifs, supporting the idea that pre-mRNA introns can be highly evolved molecules with functional and structural constraints (Deshler and Rossi 1991).

To confirm the theoretical predictions, we studied the structure and stability of *S. cerevisiae* I2, represented by a 37-nt I2s oligonucleotide encompassing the predicted hairpin. The NMR data indicated that I2s formed a hairpin structure in solution containing a 32-nt double-helical stem and a 5-nt UAACA apical loop containing the last 4 nt of the BS (Fig. 2). The stem comprised an AU-rich tract of 10 base pairs interrupted by a CGUU:GGAA internal loop that was separated from the apical loop by two Watson–Crick pairs. The UV-thermal denaturation experiments showed that this intron 2 hairpin had surprisingly low thermal stability. It is well established that both the BS and the PPT of the introns need to be single-stranded to allow binding of the BBP (SF1 in mammals) and Mud2 (U2AF_65_) proteins in the first steps of spliceosome assembly (Gahura et al. 2011; Pérez-Valle and Vilardell 2012). It is therefore remarkable that two of the BS nucleotides (A_45_ and C_46_) and the complete PPT of I2 are contained in the base-paired stem of the I2 hairpin. This observation, together with the UV thermal denaturation and NMR spectroscopy findings showing that the structure was thermally labile, supported a regulatory role for the I2 hairpin on splicing.

To assess how important I2 hairpin formation was for *SUS1* function and regulation, four structural mutants were analyzed: I2-mut1 (containing an I2 hairpin with a shortened double-helical stem), I2-mut2 (with a destabilized stem), I2-mut3 (stabilized stem), and I2-mut4 (with an intact stem and an opened apical BS loop) (Figs. 3, 4). The phenotypes observed when partially deleting the stem of the I2 hairpin (I2-mut1) suggested that this stem–loop structure was important for *SUS1* expression. However, since part of the PPT was deleted we cannot exclude the possibility that this could have affected *SUS1* splicing. Notably, I1 retention increased with the I2-mut1 mutation (Fig. 3C). Previous studies proposed that the upstream intron of *SUS1* modulated the processing of I2 (Hossain et al. 2011). Our results demonstrate that intrinsic features of I2 can also lead to I1 retention, suggesting that the downstream intron affected splicing of its upstream counterpart.

The most drastic effects were observed with the I2-mut3 mutant. The corresponding I2-mut3s oligomer formed a highly stable structure with a melting temperature more than two times higher than that of the wild-type I2s hairpin. This mutation completely inhibited I2 splicing, likely due to the inability of the spliceosome components to act in this environment. The mutants that completely destabilized the double-helical stem of the I2 hairpin (I2-mut2) or further opened the apical BS loop (I2-mut4) did not provoke a detectable increment of I1 or I2 retention, but behaved differently in terms of the amount of fully spliced *SUS1* mRNA generated. The I2-mut4 transcripts showed a slight increase of fully spliced *SUS1* mRNA relative to wild type, while a small reduction was observed in the case of I2-mut2. The small positive effect of I2-mut4 may be attributed to the marginally greater accessibility of BS nucleotides in this sequence. This would be consistent with the NMR analyses of the wild-type hairpin, which indicated the presence of only two stable base pairs in this region (Fig. 2), and with the slight reduction in melting temperature (4°C) observed for I2-mut4s relative to the wild-type oligomer (Fig. 2; Supplemental Fig. S4C). Like I2-mut1, I2-mut2 was detrimental for splicing probably because the presence of the lower double-helical stem of the I2 hairpin increases the accessibility of BS and 3′SS nucleotides to splicing factors. However, the slight reduction of fully spliced mRNA observed for I2-mut2, where the double-helical stem of the hairpin was disrupted, was much smaller than that observed for I2-mut1, containing a shortened stem. It is worth noting in this regard that in the I2-mut2 sequence an artificial PPT upstream of the BS was generated (Fig. 4A), which could promote splicing (Kupfer et al. 2004). Thus, positioning the BS in between two strong PPTs may have compensated the splicing defects caused by the destabilization of the I2 hairpin, explaining the smaller effect observed for I2-mut2 relative to I2-mut1.

The functional relevance of the I2 hairpin was further studied by analyzing the ability of the I2-mut1 and I2-mut3 mutants to accomplish Sus1 protein functions. The effect of these mutations on deubiquitination and mRNA export (Fig. 5) suggests that the reduced amount of Sus1 protein likely produced by these mutants is still enough to partially complement Sus1 functions, including *sus1*Δ temperature sensitive growth defect (data not shown). This is interesting, since overexpression of Sus1 has been shown to largely impair its functionality (Cuenca-Bono et al. 2011; Hossain et al. 2011).

In conclusion, we have demonstrated the existence of a weakly stable, 37-nt hairpin structure formed by the second intron of *S. cerevisiae SUS1* that contains BS nucleotides in its apical loop and 3′SS nucleotides immediately after the 3′ terminus of the stem. Our study shows that this structure regulates the expression of *SUS1* isoforms by influencing *SUS1* splicing. The effect of different mutants modulating the structure and stability of the hairpin suggests that this I2 stem–loop likely increases the accessibility of BS and 3′SS nucleotides to the splicing components by maintaining the BS in a readily accessible and easily opened apical loop environment and the 3′SS nucleotides unstructured immediately after the double-helical stem. In this way, the efficiency of I2 removal would be regulated by the formation of the structure.

Moreover, the effect of mutants I2-mut1 and I2-mut2 on I1 retention indicates that the I2 hairpin also modulates the splicing of the upstream intron. In this regard, it may be possible that the weakly stable hairpin has evolved as a mechanism to coordinate removal of I1, whose splicing is very inefficient and needs more time to occur. On the other hand, exon 2 skipping was promoted by mutant I2-mut1 under standard conditions. *SUS1* exon 2 skipping was previously detected in wild-type cells when different components of the splicing machinery (*MSL1*, *LEA1*, and *MUD2*) were deleted, and was explained by the role of these factors in recognizing the suboptimal BS of I1 (Hossain et al. 2009; Will and Lührmann 2011). Interestingly, our results indicate that the presence of these factors is not sufficient to supress exon skipping when intron 2 is mutated. This opens the possibility that I2-dependent mechanisms also contribute to regulate exon skipping.

## MATERIALS AND METHODS

### Sequences and structure prediction of *SUS1*

The sequences of the second intron of *SUS1* across different species of yeast were obtained from the Yeast Genome Database (SGD) and NCBI (http://www.ncbi.nlm.nih.gov/). The secondary structure predictions were carried out with the MFOLD web server (http://mfold.rna.albany.edu/?q=mfold/rna-folding-form) (Zuker 2003), and the structures were drawn using VARNA (http://varna.lri.fr/) (Darty et al. 2009).

### Large-scale RNA transcription

The wild-type I2s and mutant I2-mut2s, I2-mut3s, and I2-mut4s RNA oligomers used for NMR spectroscopy, gel electrophoresis, and UV thermal experiments were prepared by T7-polymerase in vitro transcription using synthetic oligonucleotide DNA templates. The RNA transcripts were purified on denaturing gels containing 20% acrylamide and 8 M urea. After electroelution from the gel, the RNAs were ethanol-precipitated two times and desalted with Sephadex G-25 cartridges. The I2s NMR samples were microdialyzed in aqueous solutions containing 2 mM sodium phosphate (pH 6.0) and 0.1 mM EDTA with no added salts or additionally containing 100 mM NaCl. The RNA concentration in these samples ranged between 0.10 and 0.15 mM.

### NMR spectroscopy

NMR spectra were acquired on 600 MHz (cryoprobe-equipped) and 500 MHz Bruker Avance III spectrometers, and analyzed using Topspin 1.3 (Bruker Biospin) and Sparky 3.110 (T.D. Goddard, D.G. Kneller, UCSF USA, 2004). The I2s systems were studied using two-dimensional WATERGATE-NOESY (with 150–400 ms mixing times) and WATERGATE-TOCSY experiments (60-msec mixing time) recorded in 90% H_2_O/10% D_2_O at several temperatures (between 7°C and 17°C). TOCSY and NOESY (250 msec) experiments were also acquired in D_2_O at 13°C and 23°C. The recycle delay was 2 sec in all cases.

### UV thermal denaturation

The thermal stability of wild-type I2s and mutant I2-mut2s, I2-mut3s, and I2-mut4s oligomers was monitored by measuring the UV absorbance at 260 nm as a function of temperature in a Varian Cary 100 UV/VIS spectrophotometer. The temperature was raised from 0°C–10°C to 70°C–100°C at a gradient of 0.5°C–2.0°C min^−1^ and subsequently decreased at the same rate to evaluate the reversibility of the process. The experiments were carried out using 0.4–0.5 ODU/mL of RNA (1.0–1.3 µM). The thermal denaturing profiles of the wild-type I2s sequence were examined in the following ionic conditions: 2 mM sodium phosphate (pH 6.0) with no additional salts, or containing either 100 mM NaCl or 2 mM MgCl_2_. The mutant oligomers were studied in 2 mM sodium phosphate (pH 6.0) and 100 mM NaCl. All melting experiments were repeated at least two times in each ionic condition. Before each experiment, RNA samples were heated at 95°C for ∼5 min and immediately placed on ice for 5 min.

### Gel electrophoresis

These experiments were used to assess the strand stoichiometry of the structures formed by the I2 oligonucleotides, as well as to evaluate the number of structural species formed by each sequence in a given condition. Native gels were run at 4°C for ∼14 h under constant voltage (90 V). We used 20% 19:1 acrylamide:bisacrylamide gels and 89 mM Tris–Borate (TB) as running buffer. These experiments involved 13–18 µM wild-type or mutant I2s samples, previously annealed as specified above in the following ionic conditions: 2 mM sodium phosphate (pH 6.0) with no additional salts, or containing either 100 mM NaCl or 2 mM MgCl_2_. All gels were stained with methylene blue and destained with water.

### Generation of *SUS1* constructs

All *SUS1* gene constructs contained the last 20 nt of 5′UTR. The I2 mutants (mut1, mut2, mut3, mut4, and mut1-I1Δ,) were constructed by the fusion PCR method (Yon and Fried 1989; Lesser and Guthrie 1993), using the primers specified in Supplemental Table S1. The constructs were cloned into a modified pRS425 vector containing the glyceraldehyde-3-phosphate dehydrogenase (GPD) promoter and the *CUP1* gene as a reporter without ATG, followed by the first 200 nt of the *SUS1* gene 3′UTR (see the scheme in Supplemental Fig. S5). Taq DNA polymerase (Roche) was used to amplify the WT, I2-mut1, I2-mut2, I2mut3, and I2-mut4 constructs of *SUS1*. Due to the presence of a high GC content in I2-mut3, the Q5 Hot Start High-Fidelity DNA Polymerase (New England Biolabs) was used to obtain this construct.

### Yeast cultures and microbiological techniques

Copper resistance assays were carried out by growing the transformed *cup1*Δ cells at 30°C on synthetic selective medium (SC: glucose 2%, ammonium sulphate 0.5%, yeast nitrogen base 0.17%, and supplements [Dropout]) lacking Leucine (Leu) to 0.4–0.5 OD_600_. Subsequently, 10-fold serial dilutions of an equal number of cells were made and drops spotted onto SC-leu plates containing different concentration of CuSO_4_ (Lesser and Guthrie 1993)_._ Plates were photographed after 3–5 d of incubation at 30°C. Yeast cell transformations were done by the LiAc/SS carrier DNA/PEG method (Gietz and Schiestl 2007).

### RNA extraction, reverse transcription PCRs, and semi-q-RT-PCRs

Total RNA was harvested from *sus1*Δ cells transformed with the CUP1 plasmids bearing SUS1g, SUS1-I2-mut1, SUS1-I2-mut2, SUS1-I2-mut3, or SUS1-I2-mut4 by the Hot/Acid-phenol method (Schmitt et al. 1990), and quantified using a Nanodrop. RNA quality was checked by 1% agarose gels dyed with ethidium bromide (EtBr). The cells were grown in 100 mL of SC-Leu at 30°C until 0.4 OD_600_ and then divided into two equal aliquots, in order to incubate the cell cultures under two different conditions: the cells of the first aliquot were grown 2 h more at 30°C in SC-Leu and the cells of the second aliquot were collected by centrifugation, resuspended in equal volume of preheated 42°C SC-Leu media and incubated for 20 m at 42°C. A 500 ng of DNAse I-treated RNA was used to perform the reverse transcription PCR in each case. Reverse transcription was carried out using standard procedures, with random hexamers and M-MLV reverse transcriptase (Invitrogen). For semiquantitative RT-PCR, a specific pair of primers located upstream exon 1 and exon 3 was used to amplify the transcripts of *SUS1* (25 cycles to quantify the mRNA and 35 cycles to amplify low abundant transcripts) and *SCR1* (20 cycles). *SCR1* levels are commonly used as loading control. The amplified products were run in a REALSAFE (Real laboratory) stained 3% agarose gel and visualized with a BioRad UV CCD Camera. In all cases, negative controls that included all reagents except cDNA were included. The mRNA concentrations were normalized relative to *SCR1*, and the accumulation of mRNA is represented relative to wild type. The mRNA bands were quantified by the ImageJ program (http://rsbweb.nih.gov/ij/)

### In situ hybridization (FISH)

Fluorescent in situ hybridization (FISH) against poly(A)^+^ RNA was done as described by Cuenca-Bono et al. (2011); yeast cells were grown in 100 mL of SC-Leu medium at 30°C to an 0.3 OD600. Then, cultures were rapidly shifted to 37°C incubator for 2 h. After hybridization, slides were mounted using VECTASHIELD Mounting Medium with DAPI. Detection of Cy3-oligo(dT) was performed using a Leica DM600B fluorescence microscope. mRNA accumulation was represented as the percentage of cells showing a bright signal in the nucleus that colocalized with DAPI staining (DNA). The percentages correspond to averages of at least three biological replicates.

### Total protein extraction for histone modification detection and Western blot

For the Western blot assays, the transformed *sus1*Δ cells at 30°C were grown on 50 mL synthetic selective medium lacking Leucine (SC-Leu) to 0.5 OD_600_, then the cells were harvested and washed by 20% TCA, after nitrogen freezing the cells were thawed and resuspended in 0.5 mL of 20% TCA. Cells were disrupted by glass beads and the lysate was recovered. After centrifuging the pellet during 10 min (300 rpm) the pellet was resuspended in 200 µL of 1× LB plus 50 µL of 2 M unbuffered Tris and then boiled during 3 min at 95°C. The suspension was centrifuged during 5 min at 3000 rpm and the supernatant was recovered. The proteins were separated by 15% SDS–PAGE and electrotransferred to nitrocellulose membranes, as previously described (Thiriet and Albert 1995). Membranes were stained with Ponceau S, as a transferring control, and were incubated with specific antibodies: α-H2B ubiquitinated at position 123 (Cell Signaling) and α-PGK1 (Thermoscientific). Proteins were detected with horseradish peroxidase-conjugated anti-rabbit secondary antibodies and ECL Advanced reagents (GE Healthcare). Specific signals were quantified by the ImageJ program (http://rsbweb.nih.gov/ij/).

## SUPPLEMENTAL MATERIAL

Supplemental material is available for this article.

## Supplementary Material

Supplemental Material
