# Supplementary material for: An intronic RNA structure modulates expression of the mRNA biogenesis factor Sus1
Source: RNA. 2016 Jan;22(1):75–86. doi: 10.1261/rna.054049.115 (PMC4691836; doi:10.1261/rna.054049.115)
Supplement: Supplemental Material [file supp_22_1_75__index.html]

Supplemental Material 

# An intronic RNA structure modulates expression of the mRNA biogenesis factor Sus1

## Supplemental Material

**Files in this Data Supplement:**

- Supp Fig S1.pdf
- Supp Fig S2.pdf
- Supp Fig S3.pdf
- Supp Fig S4.pdf
- Supp Fig S5.pdf
- Supp Table S1.pdf
- Supp Legends.docx
