# Supplementary figures and images for: An intronic RNA structure modulates expression of the mRNA biogenesis factor Sus1
Source: RNA. 2016 Jan;22(1):75–86. doi: 10.1261/rna.054049.115 (PMC4691836; doi:10.1261/rna.054049.115)

**A**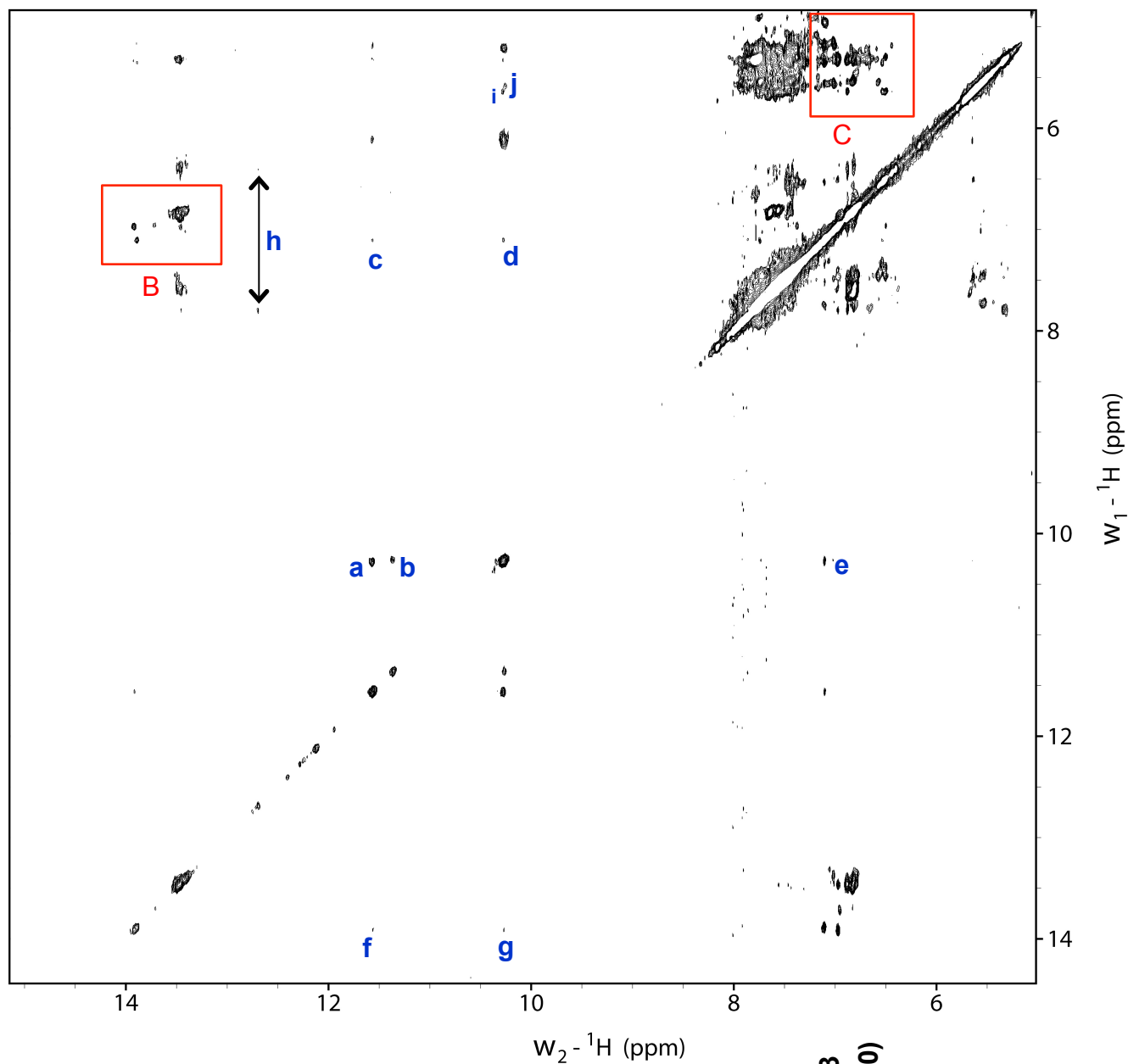**B**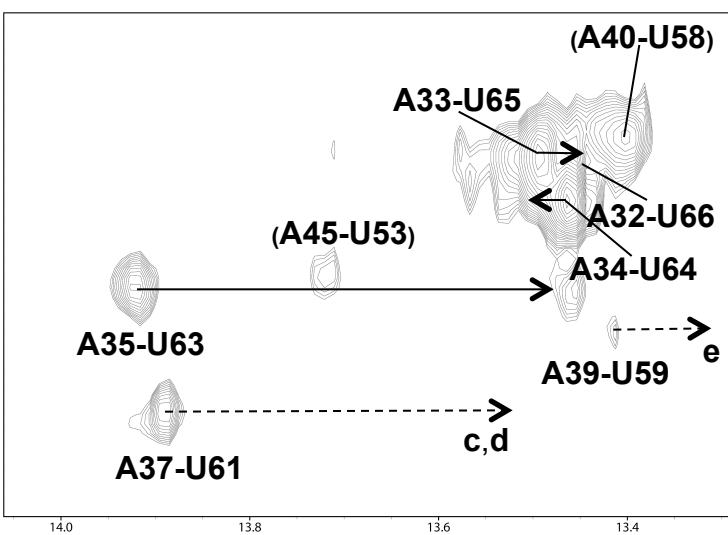**C**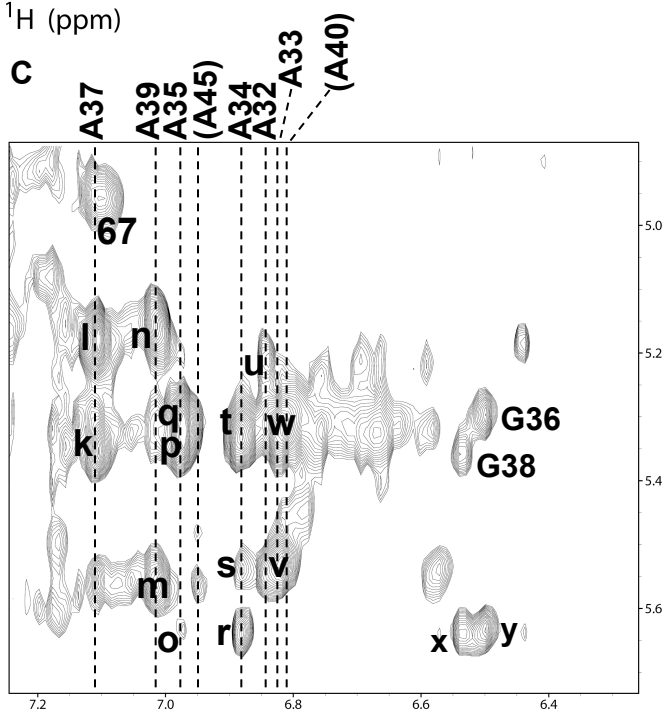

**Figure S1**  
AbuQattam et al

Supplement: Supplemental Material [file supp_054049.115_FigS1.pdf]

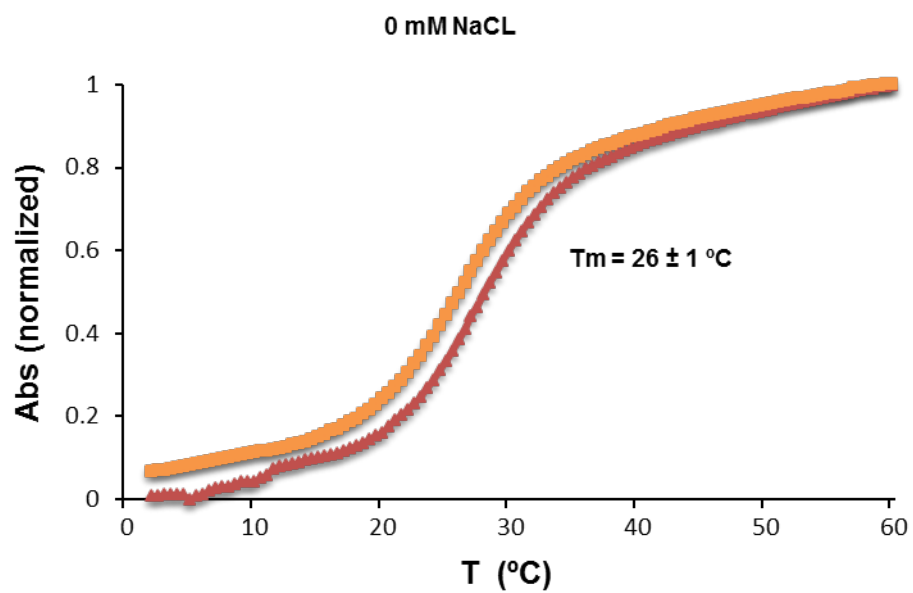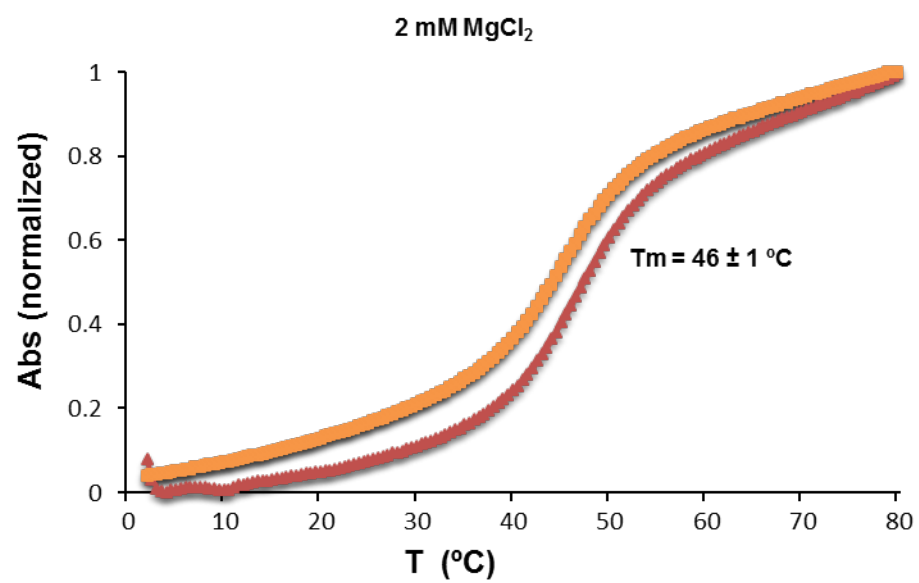

Figure S2  
AbuQattam *et al*

Supplement: Supplemental Material [file supp_054049.115_FigS2.pdf]

1- pACT1-CUP1

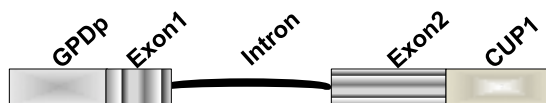

2- pSUS1-WT-I1 $\Delta$ -CUP1

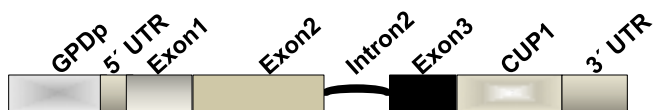

3- pSUS1-I2-mut1-I1 $\Delta$ -CUP1

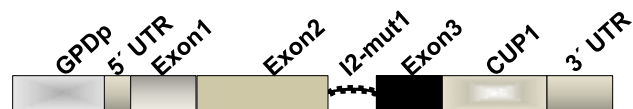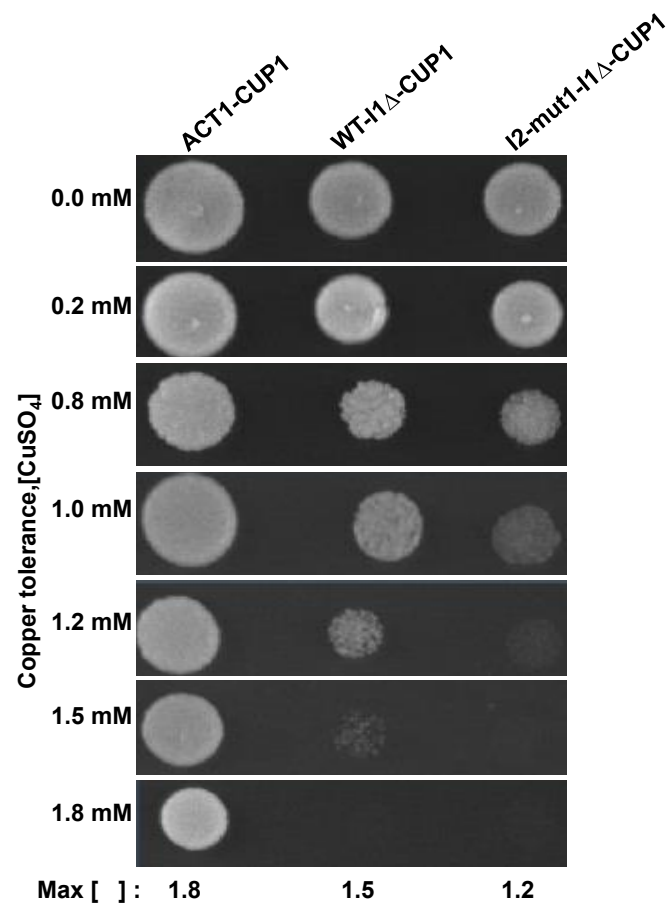

Figure S3  
AbuQattam *et al*

Supplement: Supplemental Material [file supp_054049.115_FigS3.pdf]

**A**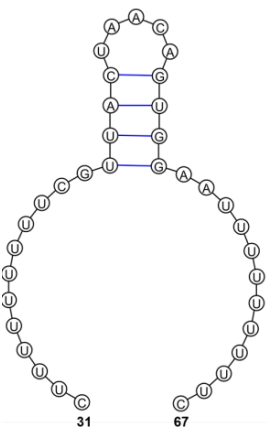**B**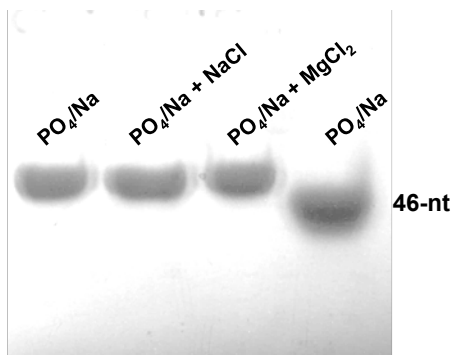**C****I2-mut2s**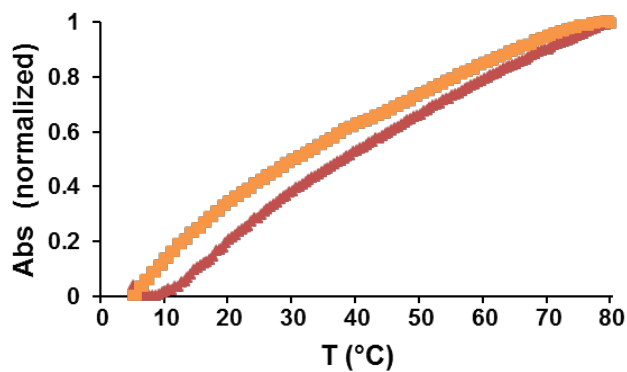**I2-mut3s**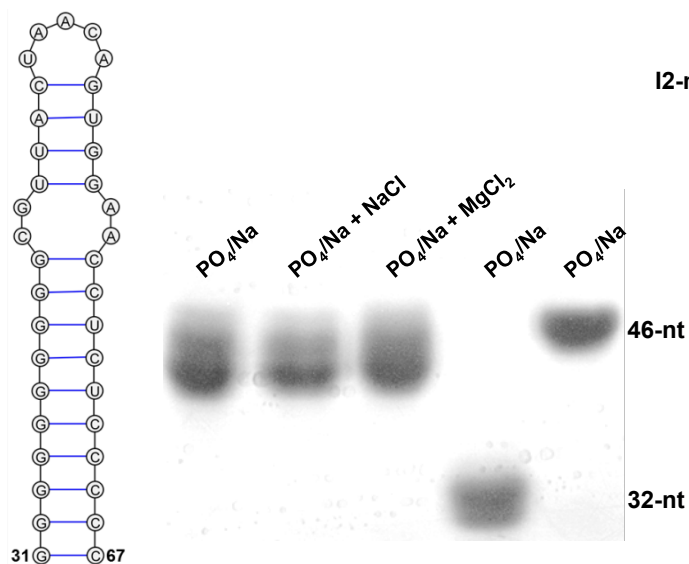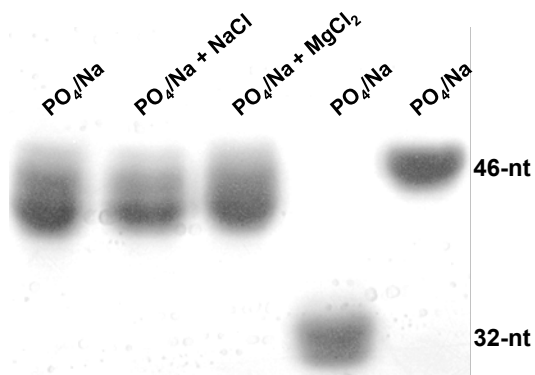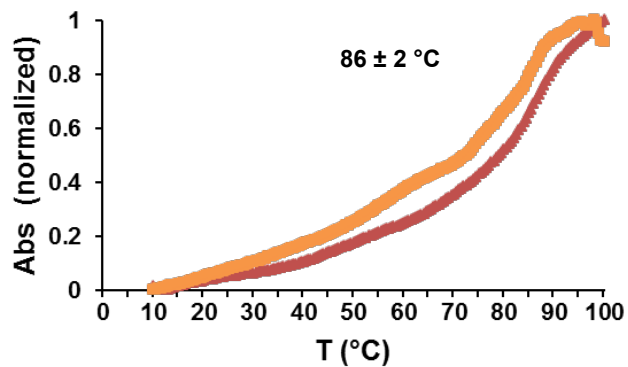**I2-mut4s**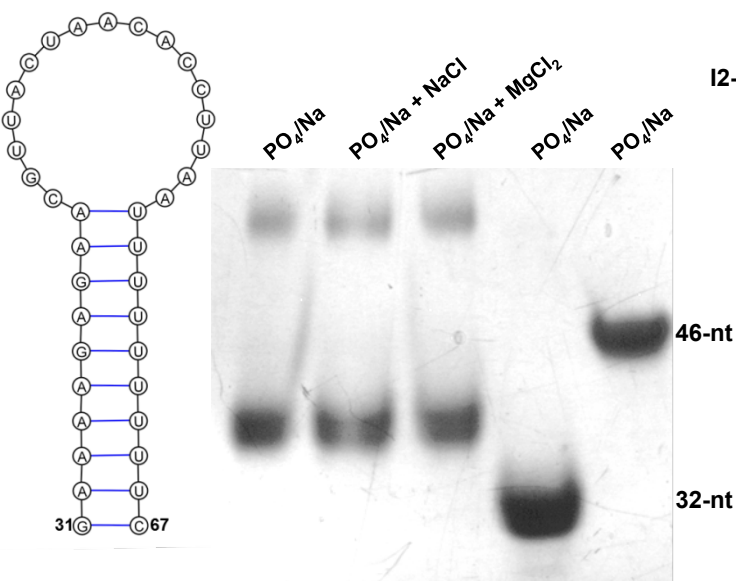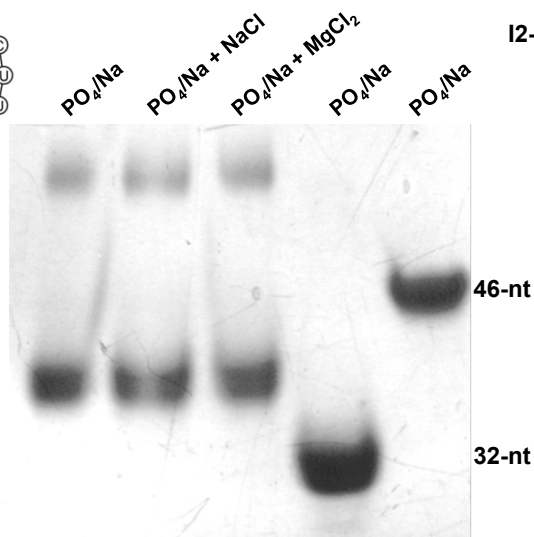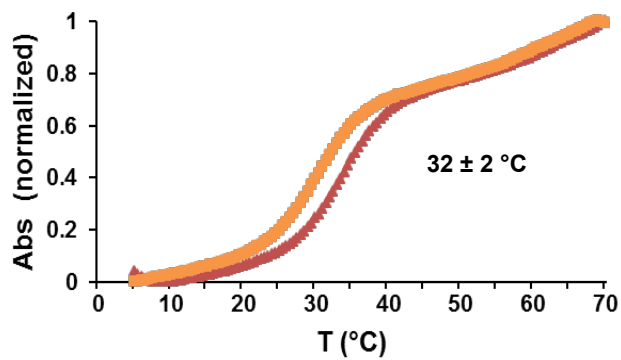

**Figure S4**  
AbuQattam *et al*

Supplement: Supplemental Material [file supp_054049.115_FigS4.pdf]

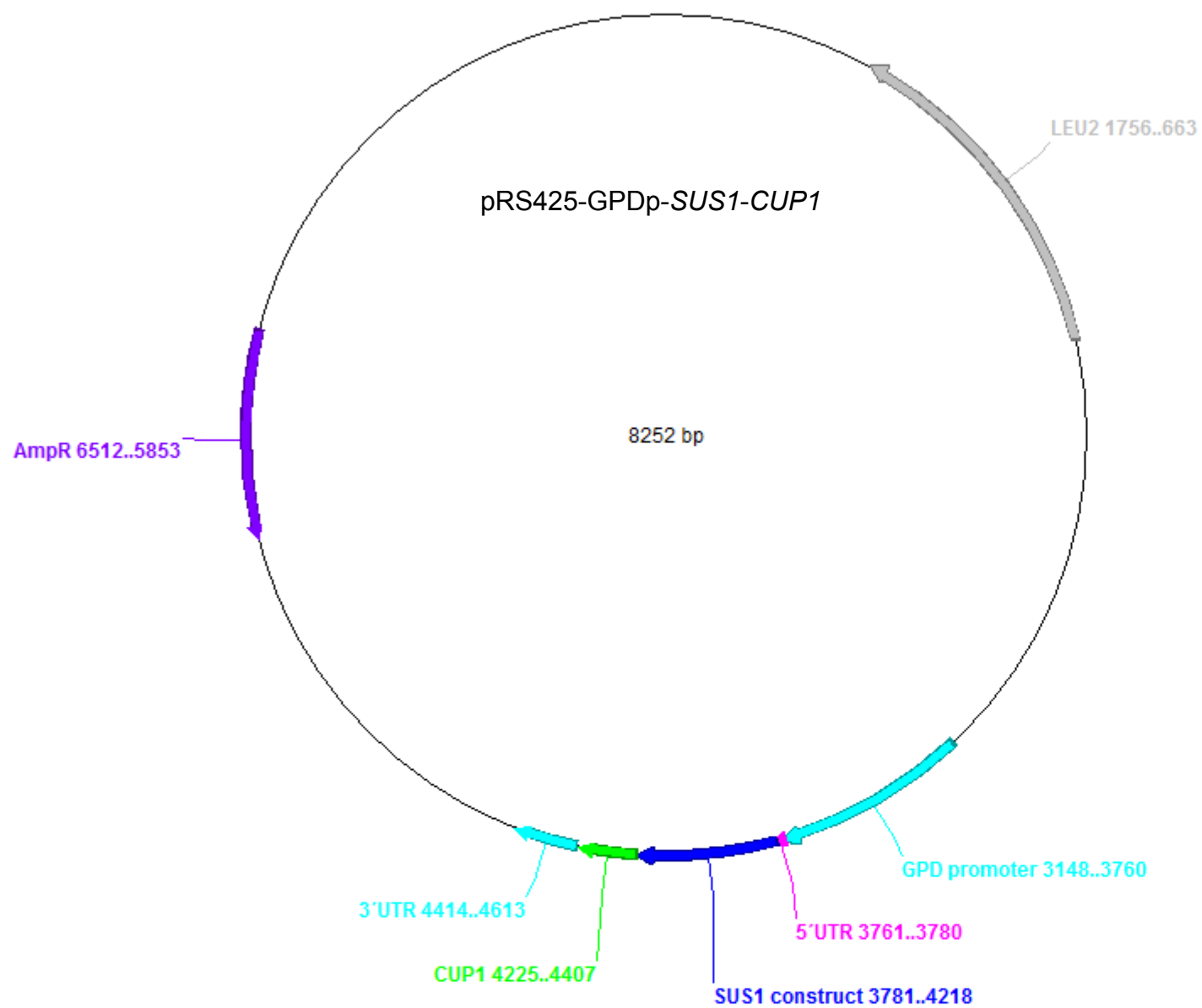

**Fig S5**  
**AbuQattam et al**

Supplement: Supplemental Material [file supp_054049.115_FigS5.pdf]
