# Supplementary material for: An intronic RNA structure modulates expression of the mRNA biogenesis factor Sus1
Source: RNA. 2016 Jan;22(1):75–86. doi: 10.1261/rna.054049.115 (PMC4691836; doi:10.1261/rna.054049.115)
Supplement: Supplemental Material [file supp_054049.115_TS1.pdf]

Table S1

Mut1 forward	CCGTAGAACCCAAAGCATTAGGTATGTGAATATTTTATCACTAAATTTATTGAAATACTAACAGTGTTTCTAG
Mut1 Reverse	GTATGTGAATATTTTATCACTAAATTTATTGAAATACTAACAGTGTTTCTAGAAATGGTATCGGATTCAACA
Mut2 forward	CACTAAATTTATTcttttttttttCGTTACTAACAG
Mut2 Reverse	ATCACTAAATTTATTcttttttttttCGTTACTAACAGTGG
Mut3 forward	ATTGggggGgGggCGTTACTAACAGTGGAAccUcUccccCTAGAAATGGTATCGGATTCAACActgcag
Mut3 reverse	CACTAAATTTATTGggggGgGggCGTTAC
Extreme forward 5´ primer for all constructs	AAAAAAaagctt<u>CAATTCTGGCCTTCACTCCA</u>ATGACTATGGATACTGC
Extreme reverse 3´primer for all constructs	AAAAAActgcagTTGTGTATCTACAATCTC
SCR1Forward	AACCGTCTTTCCTCCGTCGTAA
SCR1 reverse	AGAACTACCTTGCCGCACCA
