# Supplementary material for: An intronic RNA structure modulates expression of the mRNA biogenesis factor Sus1
Source: RNA. 2016 Jan;22(1):75–86. doi: 10.1261/rna.054049.115 (PMC4691836; doi:10.1261/rna.054049.115)
Supplement: Supplemental Material [file supp_054049.115_SuppLegends.docx]

**SUPPLEMENTARY MATERIAL**

**Figure S1**. **NMR spectroscopy analysis of *SUS1* I2s construct**. (A) Assignment of the imino region of the NOESY spectrum of I2s (H_2_O/D_2_O, 300 ms mixing time, 7 ^o^C, 100 mM NaCl). Selected crosspeaks (a) to (h) are assigned as follows: a, G36 H1-U62 H3; b, G38 H1-U60 H3; c, A37 H2-U62 H3; d, A37 H2-G36 H1; e, G38 H1-A39 H2; f, U63 H3-U62 H3; g, U63 H3-G36 H1; h, C46 H4-G52 H1; i, A37 H1’-G36 H1; j, A39 H1’-G38 H1. (B) Inset of (A) showing NOE interactions between adenine H2 resonances and uracil imino H3 protons. H2-H3 crosspeaks indicating Watson-Crick A-U base pairs are labelled, sequential cross-strand interactions are indicated with solid horizontal arrows, and crosspeaks (c), (d) and (e) are identified in (A). (C) Inset of (A) illustrating the NOE interactions established by A-U pair H2 protons in the aromatic to H1’/H5 spectral region. A-U H2 resonances are indicated with dashed lines, intraresidue H1´-H6/H8 crosspeaks are labelled with residue name and number, and pyrimidine H5-H6 crosspeaks are labelled with residue number. Crosspeaks (k) to (y) are assigned as follows: k, G38 H1’-A37 H2; l, U62 H1’-A37 H2; m, A40 H1’-A39 H2; n, U60 H1’-A39 H2; o, A35 H1’-H2; p, U64 H1’-A35 H2; q, G36 H1’-A35 H2; r, A35 H1’-A34 H2; s, A34 H1’-H2; t, U65 H1’-A34 H2; u, C67 H1’-A32 H2; v, A34 H1’-A33 H2; w, U66 H1’-A33 H2. Crosspeaks (x) and (y) are sequential interactions: x, A37 H1’-G38 H8; y, A35 H1’-G36 H8. In (B) and (C), parentheses indicate tentative assignments.

**Figure S2. UV-monitored thermal denaturation curves of I2s**. The curves show I2s melting in aqueous buffers containing 2 mM sodium phosphate (pH 6.0) with no added salts, or additionally containing 2 mM MgCl_2_. The average melting temperatures measured for I2s under these ionic conditions are indicated in the graphs. The melting curves were monophasic in all cases.

**Figure S3. The absence of *SUS1* intron 1 did not change the effect of I2-mut1 on *SUS1* processing.** Copper assay of *cup1∆* cells transformed with plasmids containing SUS1-I1*∆* -CUP1, SUS1-I2-mut1-I1*∆* -CUP1 and ACT1-CUP1 as a control. Maximum copper tolerance is indicated.

**Figure S4. *In vitro* analyses of intron 2 mutant oligonucleotides I2-mut2s, I2-mut3s and I2-mut4s.** (A) MFold-predicted secondary structures of mutant I2s oligonucleotides. (B) Native gels comparing the electrophoretic mobility of 16-20 μM mutant I2s oligonucleotide samples previously annealed in different ionic conditions: (1) 2 mM sodium phosphate (pH 6.0), (2) 2 mM sodium phosphate and 100 mM NaCl, (3) 2 mM sodium phosphate and 2 mM MgCl_2_. The right lanes contain 32-nt and/or 46-nt RNA hairpin controls. (C) UV-monitored thermal denaturation curves of mutant I2s oligomers in 2 mM sodium phosphate (pH 6.0) and 100 mM NaCl. The average melting temperatures of the I2-mut2s, I2-mut3s and I2-mut4s constructs under these ionic conditions are indicated in the graphs.

**Figure S5. Schematic representation of the modified pRS425 plasmid used for cloning the GPDp-*SUS1-CUP1* constructs.** The representation was generated by the ApE plasmid editor**.**

**Table S1. List of primers used in this study.**
